# Risk factors for acute kidney injury following orthotopic liver transplantation: the impact of changes in renal function while patients await transplantation

**DOI:** 10.1186/1471-2369-11-30

**Published:** 2010-11-08

**Authors:** Jose I Iglesias, John A DePalma, Jerrold S Levine

**Affiliations:** 1 Department of Medicine subsection of Nephrology, UMDNJ School of Osteopathic Medicine, Stratford, NJ, 08084, USA; 2 Department of Medicine subsection Nephrology, Jersey Shore University Medical Center and Robert Wood Johnson School of Medicine New Brunswick, NJ, USA; 3 Nephrology Wake Forest University Baptist Medical Center Department of Medicine, Winston-Salem, NC, 27106, USA; 4 University of Illinois at Chicago, Chicago, IL, 60612, USA; 5 Section of Nephrology, Dept. of Medicine, Jesse Brown Veterans Administration Hospital, Chicago, IL, 60612, USA

## Abstract

**Background:**

Acute kidney injury (AKI) occurs commonly in the setting of orthotopic liver transplantation (OLT). To date, the correlation between AKI post-OLT and pre-operative changes in renal function has not been rigorously examined.

**Methods:**

To determine the impact of pre-OLT changes in renal function on AKI post-OLT, as well as to identify risk factors for AKI, we analyzed the prospectively maintained NIDDK Liver Transplantation Database, which includes patients who received their first OLT between April 15, 1990, and June 30, 1994. We used the AKI Network definition of AKI.

**Results:**

Surprisingly, univariate analysis revealed that worsening renal function while awaiting OLT was protective to the development of AKI post-OLT. Independent predictors of AKI were increased body mass index, increased Childs-Pugh-Turcott score, decreased urine output during cross-clamp, improved renal function while awaiting OLT, increased post-operative stroke volume, non-Caucasian race, and post-operative use of tacrolimus.

**Conclusions:**

The correlation between improving renal function pre-OLT and AKI post-OLT may represent true protection (via ischemic pre-conditioning) or, alternatively, a masking of milder forms of AKI (via improved renal perfusion through correction of the cirrhotic milieu). These results highlight the complex interaction between liver and kidney disease, and suggest that not only the etiology but also the course of pre-OLT renal dysfunction may be a critical determinant of renal function post-OLT.

## Background

Acute kidney injury (AKI) occurs commonly in the setting of orthotopic liver transplantation (OLT). The incidence varies widely, from 12% to 80%, depending on the definition of AKI[1-4]. While awaiting OLT, patients often develop varying degrees of renal impairment, ranging from prerenal azotemia to hepatorenal syndrome and acute tubular necrosis. Although a majority of studies has identified a pre-operative elevation of serum creatinine (SCr) as a risk factor for the development of AKI post-OLT, the timing and definition of AKI have been imprecise and inconsistent[1,4-6].

Transient changes of SCr in patients awaiting OLT most often occurs as a result of changes in renal perfusion. These changes, which are attributable to events and/or interventions such as large-volume paracentesis, diuretic therapy, sepsis, or gastrointestinal bleeding, tend to reduce the predictive power of pre-OLT SCr[7,8]. To date, the correlation between the development of AKI post-OLT and pre-operative changes in renal function, as assessed by SCr, blood urea nitrogen (BUN), or estimated glomerular filtration rate (eGFR), has not been rigorously examined.

To determine the impact of pre-OLT changes in SCr, BUN, and eGFR on development of AKI post-OLT, as well as to identify additional potential risk factors for AKI, we performed an analysis of the prospectively maintained National Institute of Diabetes and Digestive and Kidney Diseases (NIDDK) Liver Transplantation Database (LTD)[9]. Given the previous lack of consistency among studies in terms of criteria and timing of AKI, we used the Acute Kidney Injury Network (AKIN) definition of AKI[10].

## Methods

### Study population

The NIDDK-LTD was established to study the demographics, clinical and laboratory characteristics, and outcomes of patients evaluated for and undergoing OLT. The NIDDK-LTD contains extensive pre- and post-OLT data for 916 recipients from 3 clinical centers: Mayo Clinic, Rochester, Minnesota; University of Nebraska Medical Center, Omaha, Nebraska; and University of California, San Francisco, California. The NIDDK-LTD is maintained by the Epidemiology Data Coordinating Center at the University of Pittsburgh Graduate School of Public Health. Data collection for the NIDDK-LTD was conducted under protocols approved by the Committees on Human Research at all three participating clinical centers. Patient recruitment, which began on April 15, 1990, and ended on June 30, 1994, included all patients who received their first OLT during this interval. Prospective data on patients were collected using standardized data collection forms at initial evaluation, OLT, and regular intervals post-OLT. Information on the specific data collected from each patient has been published previously [9]. Although data were obtained during the 1990's, the range and depth of these prospectively obtained data still offer important clinical and pathophysiologic insight into the development and risk factors of AKI in patients with severe liver disease undergoing OLT.

The current study was designed as a retrospective cohort study. Of 916 patients enrolled in the NIDDK-LTD, 688 patients were included in our analysis. We excluded 228 patients for the following reasons: age < 18 years (*n *= 138); organ transplantation in addition to OLT (*n *= 26); incomplete data (*n *= 5); and requirement for renal replacement therapy (RRT) at registration, during OLT, or on first day post-OLT (*n *= 59). Patients in the last category were excluded because the onset of AKI was felt to precede the peri-OLT period.

### Definitions

In accord with AKIN recommendations, we defined AKI as a reduction of renal function (occurring within 48 hours), manifesting by either an absolute increase of SCr ≥ 0.3 mg/dL or a percentage increase of SCr ≥ 150%. The last pre-OLT SCr was used as baseline. eGFR was determined using the 4-variable equation of the Modification of Diet in Renal Disease (MDRD) study group[11]. As urine output was only measured during the intra-operative period, we did not use urine output in defining AKI because of the unavailability of these data in the NIDDK-LTD database. Absolute changes of SCr (ΔScr), BUN (ΔBUN), and eGFR (ΔeGFR) were calculated as pre-OLT minus registration values. Percent changes of SCr (% ΔSCr), BUN (% ΔBUN), and eGFR (% ΔeGFR) were calculated by the following formula: % Δ = (pre-OLT value/registration value)-1 × 100%. Rates of change of SCr (ΔSCr/Δt_wait_), BUN (ΔBUN/Δt_wait_), and eGFR (ΔeGFR/Δt_wait_) were calculated by the following formula: rate of change = absolute change/wait list time.

### Data collection

Pre-OLT data included routine demographic variables (age, sex, race), admission physiologic variables (complete blood count, electrolytes, clotting times, albumin, liver enzymes, total bilirubin, body surface area (BSA]), body mass index (BMI]), medications, comorbidities, MELD (Model for End-Stage Liver Disease) and Childs-Pugh-Turcott scores, and renal variables (registration and pre-OLT values of BUN, SCr, and eGFR; ΔSCr, ΔBUN, and ΔeGFR; % ΔSCr, % ΔBUN, and % ΔeGFR; ΔSCr/Δt_wait_, ΔBUN/Δt_wait_, and ΔeGFR/Δt_wait_; and ^125^I-iodothalamate clearance at registration). BMI was calculated at the time of registration using actual body weight, including ascites, by the following formula: weight (kg) ÷ height (meters)^2^.

Peri- and intra-operative data included surgical and cross-clamp times; use of veno-veno bypass; administration of blood products (packed red blood cells [PRBC], platelets, fresh frozen plasma [FFP], cryoprecipitate, and colloid solutions); and hemodynamic variables (heart rate, mean arterial pressure [MAP], central venous pressure [CVP], mean pulmonary artery pressure [MPAP], pulmonary artery wedge pressure [PAWP], cardiac output [CO], stroke volume [SV], and systemic vascular resistance [SVR]; and urine output. Hemodynamic variables and urine output were determined at the start of surgery, during cross-clamp (anhepatic phase) and following cross-clamp (reperfusion phase).

### Data analysis

The primary outcome was development of AKI. As the number of patients with AKI stage 2 and stage 3 (AKI-2 and AKI-3) was too small for meaningful analysis (45 and 20 patients, respectively), all statistical analyses unless otherwise indicated were performed for patients who developed AKI (AKI) vs. those who did not (NO AKI).

Summary statistics were computed for both the AKI and NO AKI cohorts. Continuous variables were expressed as mean ± standard deviation and compared by Student t test or Wilcoxon rank-sum test. Categorical variables were compared by Fisher's exact test or chi-square analysis. Comparison of cohorts included both univariate and multivariate analysis. Variables significant by univariate analysis at p < 0.05 were candidates for multivariate analysis. We performed logistic regression analysis with forward variable selection to determine variables independently predictive of AKI. Stepwise selections for logistic regression were based on the maximum likelihood ratio. For continuous variables, the odds ratio (OR) represents the relative amount by which the probability of obtaining the outcome variable increases or decreases if the independent variable is increased by exactly one unit. OR and their 95% confidence intervals (CI) were determined by exponentiation of the regression coefficient or its upper and lower 95% CI, respectively.

## Results

### Univariate analyses of non-renal-function-associated risk factors for AKI post-OLT

To determine factors correlating with AKI following OLT, we analyzed multiple routinely available demographic, clinical, and laboratory variables obtained during the pre-, intra- and post (first 48 hours)-operative periods for 916 OLT recipients registered in the NIDDK-LTD.

A total of 688 patients were evaluated, of whom 243 (35%) developed AKI. Of these, 178 (73%) developed AKI-1, 45 (18%) AKI-2, and 20 (8%) AKI-3. 6 patients (all with AKI-3) required RRT. We compared patients who developed AKI (AKI) with those who did not (NO AKI) by univariate analysis for multiple characteristics, including pre-operative factors (Table 1), associated co-morbidities (Table 2), therapeutic interventions (Table 3), peri-operative urine outputs and operative variables (Table 4), and intra-operative hemodynamic variables (Table 5).

**Table 1:** Univariate analysis of demographic and pre-operative variables in patients with and without AKI.

	No AKI (n = 445)	AKI (n = 243)	p value
Age	48.7 ± 11.3	50 ± 10.2	0.19
Sex (male)	232 (52%)	160 (66%)	0.02
Race (Caucasian)	369 (82%)	185 (76%)	0.035
BSA (m^2^)	2.10 ± 0.30	2.26 ± 0.22	0.00001
BMI (kg/m^2 ^)	25.3 ± 4.4	28 ± 5.8	0.00001
Hgb (g/dL)	11.1 ± 1.9	11.0 ± 1.9	0.76
WBC x10^9^/L	6.4 ± 4.5	6.1 ± 3.4	0.61
Platelets x10^9^/L	137 ± 135	104 ± 67	0.00001
PT (sec)	14.9 ± 4.7	15.0 ± 2.5	0.00001
PTT (sec)	37.3 ± 12.4	40 ± 13.7	0.003
Na (mmol/L)	136 ± 5.3	135 ± 5.9	0.0005
HCO_3 _(mmol/L)	23.7 ± 3.7	24 ± 4	0.12
AST (U/L)	133 ± 144	130 ± 125	0.60
ALT (U/L)	94 ± 144	85 ± 94	0.40
Total bilirubin (mg/dL)	6.7 ± 9	7 ± 10	0.07
Albumin (g/dL)	3.1 ± 0.6	2.87 ± 0.64	0.00007

variables in patients with and without AKI.

**Table 2:** Univariate analysis of co-morbidities, hepatic diagnoses, and complications in patients with and without AKI.

	NO AKI (n = 445)	AKI (n = 243)	p value
**Registration co-morbidities**			
Coronary artery disease	15 (3%)	11 (4%)	0.52
Diabetes	64 (14%)	31 (13%(	0.64
Hypertension	69 (16%)	45 (19%)	0.33
**Hepatic disease leading to transplant**			
Alcoholic liver disease	81 (18%)	60 (25%)	0.048
Chronic hepatitis	162 (36%)	108 (44%)	0.041
Hepatitis B	28 (6%)	17 (7%)	0.74
Hepatitis C	63 (15%)	42 (18%)	0.31
Non-alcoholic steatohepatitis	160 (36%)	52 (21%)	0.00001
**Complications while on waiting list**			
Ascites (registration)	325 (73%)	195 (82%)	0.04
Ascites (pre-operative)	289 (65%)	181(74%)	0.01
Variceal bleed (any time)	142 (32%)	70(29%)	0.40
Porto-systemic shunt (any time)	33 (7%)	24 (10%)	0.26
Bacterial peritonitis (any time)	59 (13%)	44 (17%)	0.09
Porto-systemic encephalopathy (any time)	195 (44%)	141 (58%)	0.00001
**MELD score**	15.7 ± 6	15.8 ± 5	0.8
**Child-Pugh-Turcott score**	8.4 ± 1.6	9.1 ± 1.5	0.00001
**Other**			
Mechanical ventilation (post-operative)	271 (61%)	171 (70%)	0.00001
Retransplant	26 (6%)	24 (10%)	0.06

**Table 3:** Univariate analysis of effect of therapeutic interventions in patients with and without AKI.

	NO AKI (n = 445)	AKI (n = 243)	p value
**Wait list (any time)**			

Aminoglycoside	55 (12%)	24 (10%)	0.33

**Inotrope use**	367 (82%)	198 (81%)	0.75

Time of surgery	407(91%)	213(87%)	0.10

Anhepatic phase (Cross clamp)	421(95%)	228(94%)	0.73

Post operative	414(93%)	224(92%)	0.75

**Pre-operative**			

Loop diuretic	172 (38%)	108 (44%)	0.14

Spironolactone	226 (51%)	133 (53%)	0.30

**Intra-operative**			

Albumin (ml)	1059 ± 1311	1199 ± 1509	0.50

Colloid (ml)	2413 ± 2400	2428 ± 3336	0.30

Packed red blood cells (ml)	3440 ± 3281	3974 ± 3758	0.003

Platelets (ml)	813 ± 98	1082 ± 1118	0.0002

Cryoprecipitate (ml)	228 ± 490	212 ± 405	0.90

Fresh frozen plasma (ml)	3695 ± 2759	4582 ± 3121	0.00002

Venovenous bypass	270 (61%)	128 (53%)	0.044

**Post-operative**			

Loop diuretic use	334 (75%)	208 (85%)	0.001

Cyclosporin use	330 (74%)	150 (61%)	0.001

Tacrolimus use	46 (10%)	42 (17%)	0.012

Cyclosporin level, day 1	298 ± 220	269 ± 233	0.09

Cyclosporin level, day 3	816 ± 517	793 ± 548	0.75

Tacrolimus level, day 1	7.8 ± 11.0	6.2 ± 5.3	0.58

Tacrolimus level, day 3	9.2 ± 13.0	15.6 ± 18.0	0.14

**Table 4:** Univariate analysis of urine output and other operative variables in patients with and without AKI.

	NO AKI (n = 445)	AKI (n = 243)	p value
**Operative variables**			
Days on waiting list	149 ± 152	173 ± 206	0.36
Duration of surgery (hrs: mins)	6:44 ± 1:47	6:46 ± 1.58	0.80
Duration of cross-clamp (hrs: mins)	1:19 ± 0:35	1:11 ± 0:33	0.004
**Urine output (ml)**			
Surgery start	760 ± 672	810 ± 903	0.40
Cross-clamp	175 ± 186	113 ± 139	0.00001
Post-operative	785 ± 806	811 ± 877	0.60

**Table 5:** Univariate analysis of intra-operative hemodynamic factors in patients with and without AKI.

	NO AKI (n = 445)	AKI (n = 243)	p value
**Surgery start**			
Heart rate (beats per minute)	85 ± 15	86 ± 17	0.75
MAP (mm Hg)	75 ± 13	77 ± 13	0.14
CVP (mm Hg)	12.6 ± 5.4	12.7 ± 5.2	0.76
MPAP (mm Hg)	19 ± 5.8	20.6 ± 8.0	0.17
PAWP (mm Hg)	15.3 ± 4.9	16.0 ± 5.3	0.34
CO (L/min)	8.5 ± 3.3	9.7 ± 3.7	0.00001
SV (ml)	101 ± 37	117 ± 72	0.00001
SVR (dynes)	667 ± 279	599 ± 240	0.00002
			
**Cross-clamp**			
Heart rate (beats per minute)	99.0 ± 17.0	103.0 ± 16.0	0.001
MAP	82.0 ± 12.5	84.0 ± 13.0	0.043
CVP	10.1 ± 5.4	9.3 ± 5.4	0.10
MPAP	14.3 ± 6.3	14.7 ± 7.2	0.87
PAWP	11.8 ± 4.7	11.5 ± 4.4	0.74
CO	6.9 ± 2.4	7.8 ± 7.5	0.23
SV	71.0 ± 26.9	77.8 ± 64.0	0.97
SVR	950.0 ± 415.0	970.0 ± 491.0	0.86
			
**Post-cross-clamp**			
Heart rate (beats per minute)	98.0 ± 14.0	98.0 ± 15.0	0.50
MAP	74.0 ± 10.6	75.0 ± 10.4	0.50
CVP	12.1 ± 4.6	12.3 ± 4.9	0.60
MPAP	20.0 ± 5.3	21.0 ± 7.1	0.16
PAWP	14.7 ± 4.8	15.3 ± 4.5	0.32
CO	11.0 ± 3.7	12.6 ± 3.7	0.00001
SV	114.0 ± 38.0	134 ± 77	0.00003
SVR	482.0 ± 167.0	432.0 ± 161.0	0.00003

Abbreviations: CO, cardiac output; CVP, central venous pressure; MAP, mean arterial pressure; MPAP, mean pulmonary artery pressure; PAWP, pulmonary artery wedge pressure; SV, stroke volume; SVR, systemic vascular resistance.

The following pre-operative variables were significantly associated with the development of AKI (Table 1): male sex (p = 0.02), non-Caucasian race (p = 0.035), increased BSA (p < 0.00001), increased BMI (p < 0.00001), decreased platelet count (p < 0.00001), elevated prothrombin time (p < 0.00001), elevated partial thromboplastin time (p = 0.003), decreased serum albumin (p < 0.00007), and decreased serum sodium (p = 0.0005).

The following diagnoses and co-morbidities were significantly associated with the development of AKI (Table 2): Childs Pugh Turcott score (p = 0.00001), ascites while awaiting OLT (p = 0.04) or at time of OLT (p = 0.010), post-OLT mechanical ventilation (p < 0.00001), porto-systemic encephalopathy (p < 0.00001), alcoholic liver disease (p = 0.048), chronic hepatitis (p = 0.04), and non-alcoholic steatohepatitis (p < 0.0001).

Therapeutic interventions associated with the development of AKI included (Table 3): post-OLT use of loop diuretic (p = 0.001) or tacrolimus (p < 0.0001); intra-operative administration of PRBC (p = 0.003), platelets (p = 0.0002), or FFP (p < 0.00002); lack of veno-venous bypass (p = 0.044); and lack of cyclosporine (p = 0.001).

Intra-operative urine output differed significantly for AKI patients only during cross-clamp, during which they had a decreased urine flow (p < 0.00001) (Table 4). The duration of cross-clamp was shorter in AKI patients (p = 0.004). This may reflect a decreased use of venovenous bypass in AKI patients (Table 3), since cross-clamp times were longer in AKI patients, when stratified according to use of venoveno bypass (p < 0.0001, not shown).

Hemodynamic variables were assessed at start of surgery, during cross-clamp, and after cross-clamp (Table 5). At start of surgery, AKI patients had increased CO (p < 0.00001), increased SV (p < 0.00001), and decreased SVR (p < 0.00002). During cross-clamp, AKI patients had higher heart rates (p = 0.001) and higher MAP (0.043). Finally, following release of cross-clamp, AKI patients had increased CO (p < 0.00001), increased SV (p < 0.00003), and decreased SVR (p < 0.00003).

### Univariate analyses of pre-OLT measures of renal function as risk factors for AKI post-OLT

To determine the impact of renal function on development of AKI post-OLT, we evaluated multiple absolute measures of renal function obtained at registration or pre-OLT, as well as changes in these same measures of renal function while awaiting OLT (Table 6). No correlation existed between AKI post-OLT and absolute values of SCr, BUN, and eGFR, either at registration or pre-OLT.

**Table 6:** Univariate analysis of measures of renal function in patients with and without AKI.

Renal functional parameter	NO AKI (n = 445)	AKI (n = 243)	p value
Iodothalamate clearance (ml/min) (registration)	97 ± 35	105 ± 66	0.44
SCr (mg/dL) (registration)	1.05 ± 0.60	1.05 ± 0.40	0.46
BUN (mg/dL) (registration)	19 ± 18	17 ± 14	0.16
eGFR (ml/min) (registration)	83 ± 31	84 ± 32	0.81
SCr (mg/dL) (pre-operative)	1.19 ± 0.60	1.09 ± 0.40	0.45
BUN (mg/dL) (pre-operative)	19.7 ± 17.0	20.0 ± 15.4	0.66
eGFR (ml/ml) (pre-operative)	76.0 ± 31.0	81.4 ± 33.0	0.058
ΔSCr (mg/dL)	0.13 ± 0.70	0.036 ± 0.390	0.017
ΔBUN (mg/dL)	1.08 ± 23.0	3.05 ± 21.0	0.11
ΔeGFR (ml/min)	-7.4 ± 26.0	-2.6 ± 31.0	0.021
% ΔSCr	19 ± 67	9 ± 40	0.014
% ΔBUN	58 ± 170	72 ± 181	0.74
% ΔeGFR	-3.7 ± 45.0	3.3 ± 38.0	0.014
ΔSCr/Δt (mg/dL/day) (mg/dL/day)	0.004 ± 0.034	-0.0014 ± 0.015	0.007
ΔBUN/Δt (mg/dL/day)	0.12 ± 1.30	0.166 ± 0.90	0.114
ΔeGFR/Δt (ml/min/day)	-0.15 ± 1.29	0.04 ± 0.66	0.006

Abbreviations: BUN, blood urea nitrogen; eGFR, estimated glomerular filtration rate; SCr, serum creatinine.

To convert to S.I. units: multiply BUN by 0.357 (mmol/L); multiply SCr by 88.4 (μmol/L).

Unexpectedly, univariate analysis revealed an inverse relationship between improving renal function while awaiting OLT and development of AKI post-OLT. On average, AKI patients had a greater degree of renal functional preservation, or even improvement, while awaiting OLT than did NO AKI patients. Thus, AKI post-OLT was significantly correlated with a decreased ΔSCr, a decreased % ΔSCr, and a decreased ΔSCr/Δt_wait _(ΔSCr = 0.036 ± 0.390 vs. 0.13 ± 0.70 mg/dL, p = 0.017; % ΔSCr = 9 ± 40% vs. 19 ± 67%, p = 0.014; and ΔSCr/Δt_wait _= -0.0014 ± 0.015 vs. 0.004 ± 0.034 mg/dL/day, p = 0.007). Similarly, the development of AKI was significantly correlated with a decreased ΔeGFR, a decreased % ΔeGFR, and a decreased ΔeGFR/Δt_wait _(ΔeGFR = -2.6 ± 31 vs. -7.4 ± 26 ml/min, p = 0.021; % ΔeGFR = -3.7 ± 45% vs. 3.3 ± 38%, p = 0.014; and ΔeGFR/Δt_wait _= -0.0014 ± 0.015 vs. 0.004 ± 0.034 ml/min/day, p = 0.007).

### Multivariate analysis of risk factors for AKI post-OLT

We used forward stepwise logistic regression analysis to determine which variables identified by univariate analyses (Tables 1, 2, 3, 4, 5, 6) were independent predictors of AKI post-OLT (Table 7). Independent predictors of AKI, in descending order of coefficient of determination, were increased BMI, increased Childs-Pugh-Turcott score, decreased urine output during cross-clamp, improved renal function while awaiting OLT, increased post-OLT SV, non-Caucasian race, and post-OLT use of tacrolimus.

**Table 7:** Multivariate logistic regression analysis of risk factors for the development of all AKI.

Variable	Regression coefficient	SE	p value	OR	95% CI	Coefficient of determination*
Body mass index	0.085	0.02	0.00001	1.09	1.045-1.130	0.070
Childs-Pugh-Turcot score	0.27	0.07	0.00001	1.31	1.15-1.49	0.051
Urine output (cross-clamp)	-0.002	0.001	0.003	0.99	0.996-0.990	0.032
ΔSCr/Δt (mg/dL/day)	-0.002	0.001	0.019	0.99	0.97-1.00	0.030
Stroke volume (post-operative)	0.006	0.003	0.027	1.006	1.001-1.011	0.015
Race (non-Caucasian)	0.662	0.25	0.008	1.93	1.18-3.10	0.013
Tacrolimus (post-operative)	0.662	0.29	0.022	1.93	1.1-3.4	0.011

* The cumulative coefficient of determination for all variables combined is 0.222.

### Categorical analysis of effect of changes in renal function while awaiting OLT on post-operative AKI

The emergence of improved renal function as an independent risk factor for AKI post-OLT was unanticipated. To confirm the impact of pre-operative changes of renal function on AKI post-OLT, we performed several additional analyses. In the first, we subdivided patients into those whose renal function remained the same or declined while awaiting OLT vs. those whose renal function improved (Table 8). AKI occurred in 42% of patients whose eGFR improved vs. 32% of those whose eGFR remained the same or decreased (p = 0.014, OR = 1.53, 95% CI = 1.10-2.20). A similar trend was observed in comparing patients whose SCr increased vs. those whose SCr remained the same or decreased, although the difference did not achieve statistical significance (38% vs. 32%, p = 0.11, OR = 1.30, 95% CI = 0.95-1.80).

**Table 8:** Categorical analysis of measures of renal function in patients with and without AKI.

	NO AKI (n = 445)	AKI (n = 243)	p value	OR	95% CI
Increased eGFR	130 (29%)	94 (39%)	0.014	1.53	1.10-2.20
Decreased (or unchanged) eGFR	315 (71%)	149 (61%)			

Decreased (or unchanged) SCr	227 (51%)	140 (57%)	0.11	1.30	0.95-1.80
Increased SCr	218 (49%)	103 (43%)			

**Figure 1:**
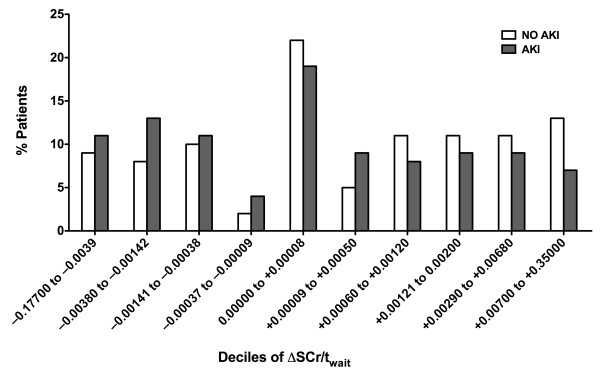
**Distribution of rates of change of serum creatinine (ΔSCr/Δtwait) while awaiting orthotopic liver transplantation (OLT) in patients who did or did not develop post-OLT acute kidney injury (AKI)**. Patients were stratified in order to according to ΔSCr/Δtwait and subdivided into deciles containing equal numbers of patients. ΔSCr/Δtwait was defined as the absolute change of serum creatine (ΔSCr) while awaiting OLT (SCr pre-OLT minus SCr at registration) divided by the time awaiting OLT. The percentage of the total number of patients (n = 243) who developed AKI (AKI) falling within each decile is plotted. Similarly, the percentage of the total number of patients (n = 445) who did not develop AKI (NO AKI) within each decile is plotted. Negative values of ΔSCr/Δtwait correspond to improved renal function, while positive values of ΔSCr/Δtwait correspond to worsened renal function. The inverse relationship between degree of renal functional improvement and risk for AKI was statistically significant (p = 0.008).

In the second analysis, we subdivided patients according to ΔSCr/Δt_wait _(Figure 1). Negative values of ΔSCr/Δt_wait _indicate improved renal function while awaiting OLT, whereas positive values indicate worsened renal function. In general, for those deciles of ΔSCr/Δt_wait _corresponding to improved renal function (ΔSCr/Δt_wait _< 0), an increased percentage of patients developed AKI. The opposite trend was observed for those deciles corresponding to stable or worsening renal function (ΔSCr/Δt_wait _≥ 0). The inverse relationship between degree of renal functional improvement and risk for AKI was statistically significant (p = 0.008).

Finally, we repeated our analysis including the 11 patients who required RRT on the first day post-OLT. These patients were excluded because the onset of their AKI was felt to precede the peri-OLT period and therefore to be independent of operative factors. It is possible, however, that their exclusion may have led to an under-representation of patients with declining renal function while awaiting OLT. Notably, even upon inclusion of these patients, a significant correlation still existed between the development of AKI and the same pre-OLT renal functional variables: namely, decreased ΔSCr and % ΔSCr; decreased ΔeGFR and % ΔeGFR; decreased ΔSCr/Δt and ΔeGFR/Δt (Additional file 1, Table S1).

### Follow up at 7 days of AKI and NO AKI patients

We examined the outcome of AKI and NO AKI patients at 7 days post-OLT. Of the 243 patients who developed AKI within 2 days of OLT, 3 died, 160 recovered, and 80 still suffered from AKI (53 AKI-1, 3 AKI-2, and 24 AKI-3). Eight patients received RRT. Of the 445 patients without AKI in the first 2 days post-OLT, 4 died and 291 subsequently developed AKI (77 AKI-1, 13 AKI-2, 60 AKI-3), with 25 requiring RRT. The etiology and risk factors for AKI developing > 2 days post-OLT are likely distinct from those for AKI in the immediate period post-OLT.

## Discussion

We performed a retrospective analysis of patients enrolled in the NIDDK-LTD to identify risk factors for the development of AKI immediately following OLT (≤ 48 hours). We examined a variety of demographic, clinical, and laboratory variables obtained at registration, pre-OLT, intra-operatively, and after OLT in 688 patients undergoing OLT between April, 1990, and June, 1994. Our most important finding is that those patients whose renal function declined while awaiting OLT, as assessed by an increased SCr or a decreased eGFR, were at decreased risk for AKI post-OLT. This seemingly paradoxical result is in contrast to previous studies, in which pre-OLT renal dysfunction was identified as a strong predictor of AKI post-OLT. In our analysis, the absolute level of renal function, as assessed by ^125^I-iodothalamate clearance, eGFR, SCr, or BUN, obtained at registration or immediately pre-OLT, did not differ between patients with and without AKI. Rather, the change in renal function in the interval from registration to OLT was the critical determinant whether OLT recipients developed AKI.

Several considerations bear on the discrepancy between our results and those of previous studies. The most important is a previous lack of consensus with respect to the definition and timing of AKI. Earlier studies varied widely in the definition and timeframe in which AKI was studied[12,13]. To circumvent these issues, we used the AKIN definition of AKI, and limited our analysis to within 48 hours following OLT.

The issue of timing is subtle but potentially critical. While AKI can occur anytime following OLT, its etiology likely varies with time from surgery. AKI occurring immediately following OLT and AKI occurring later during the post-OLT course are probably distinct entities with distinct predisposing factors and epidemiology. Moreover, as compared to the general surgical population, patients undergoing OLT have both shared and specific risk factors for AKI [13]. Our interest was in risk factors specific to OLT. We reasoned that the shorter the interval between OLT and AKI, the more likely that AKI would reflect factors particular to OLT.

In addition to issues of timing, most previous studies, identifying pre-operative renal dysfunction as a risk factor for AKI, have focused on more severe degrees of renal dysfunction. For example, renal dysfunction pre-OLT has been defined categorically as a SCr > 1.5 mg/dL, while AKI post-OLT has been defined as a requirement for RRT or a doubling of SCr[14-17]. Contreras et al showed that an elevated pre-OLT SCr was the strongest predictor of AKI requiring RRT within the first 7 days post-OLT[12]. Similarly, Sanchez et al found that a SCr > 1.9 mg/dL or a BUN > 27 mg/dL were strong predictors of AKI requiring RRT, although these authors did not indicate clearly the timeframe post-OLT that they studied[13].

In contrast, we used continuous measures of renal function pre-OLT as well as a standardized definition for post-OLT AKI. To our knowledge, no studies have evaluated post-OLT AKI by AKIN definitions, and only two studies have used the RIFLE criteria, on which the AKIN definitions are based[18]. Like AKIN, RIFLE uses three categories of graded renal dysfunction (denoted risk, injury, and failure). Earlier studies using RIFLE criteria offer some support to our conclusions. O'Reardon et al found that an increased SCr pre-OLT was a risk factor for the development of renal failure, the most severe RIFLE category[4]. Notably, an elevation of SCr pre-OLT was not a risk factor for renal injury, a less severe RIFLE category. As in our study, patients who developed renal injury had better pre-operative renal function, with lower SCr and higher eGFR. It should be noted, however, that these authors evaluated renal failure and injury at a later time, namely, two weeks post-OLT[4]. While Guitard et al, in a retrospective analysis of 100 OLT patients, also using RIFLE criteria, found that an elevated SCr pre-OLT was associated with renal failure by univariate analysis, elevated pre-OLT SCr dropped out as an independent predictor by multivariate analysis[18].

Several potential explanations may account for the apparent protective effect of pre-OLT renal functional impairment on the development of AKI post-OLT. First, AKI in these patients may have been masked by an improvement in renal perfusion, leading to increased eGFR and decreased SCr. Because OLT reverses many circulatory abnormalities associated with decreased renal perfusion [19], patients often recover renal function post-OLT. It is estimated that a majority of patients awaiting OLT have some form of reversible renal dysfunction due to diminished renal perfusion[20-23]. Thus, in our study, it is possible that pre-OLT declines in renal function were reflective of changes in renal perfusion rather than intrinsic injury or loss of renal mass. OLT, by improving renal perfusion and inducing a decline in SCr and rise in eGFR, could mask small deteriorations in renal function consistent with milder stages of AKI. In accord with this possibility is the fact that an improvement of renal function was a more powerful predictor for AKI-2/3 than for AKI-1 (Additional file 1 Tables S2, S3, and S4). Second, consideration should be given to the possibility that the pre-OLT decline in SCr among patients developing AKI is dilutional rather than indicative of improved renal function. While we cannot formally exclude this possibility, it is noteworthy that AKI patients sustained a loss, rather than gain, of weight (14.5 ± 32.0 kg) during the time from registration until OLT, making it unlikely that their decline of SCr can be attributed to dilutional effects. Third, the pre-OLT decline in SCr may reflect a loss of lean body mass, and it is the debilitation resulting from such a loss of lean body mass that predisposes to the development of AKI. Finally, pre-operative declines of renal dysfunction may truly protect patients from AKI, perhaps via ischemic preconditioning, as previously described for liver, kidney, and heart[24-26].

Other independent predictors of AKI post-OLT were increased BMI, decreased urine output during cross-clamp, increased post-OLT SV, non-Caucasian race, post-OLT use of tacrolimus, and Childs Pugh Turcott score. These factors likely reflect the severity of underlying liver disease (Childs-Pugh-Turcott score and increased post-OLT SV) and/or degree of renal ischemia (decreased urine output and post-OLT tacrolimus). Non-Caucasian race has been identified in other studies as a risk factor for AKI, for example, among patients undergoing cardiopulmonary bypass[27,28]. Other studies have noted an association between tacrolimus and renal dysfunction[29-32].

An increased BMI was the strongest independent predictor of AKI post-OLT. A larger BMI may affect dosing and volume of distribution for drugs with a potential for nephrotoxicity[33]. Obese patients may have a greater post-operative inflammatory response with increased risk of multi-organ dysfunction[34]. In addition, patients with larger BMI may receive small-for-size hepatic grafts with resultant graft dysfunction and renal hypoperfusion[35]. Finally, the possibility exists that a larger BMI reflects volume overload and profound ascites, with the observed decline of SCr in AKI patients being a dilutional effect rather than an indication of improved renal function. As discussed above, this seems inlikely, since these patients lost, rather than gained, considerable weight while awaiting OLT. However, it is important to note that the determination of BMI in patients with ascites is fraught with error, and no formula for BMI in these patients has been validated.

Our study has several strengths. The most important is our use of the NIDDK-LTD, which contains prospective data from three centers over a five-year period. Also, we evaluated risk factors for AKI over a prolonged timeframe, from registration up to and following OLT. Finally, we used a very rigorous definition of AKI, as well as continuous measures of renal function, and we limited our analysis to the initial 48 hours post-OLT.

Although data on urine output were not available for the pre- and post-OLT periods, AKI and NO AKI patients did not differ in relative weight change from immediately before OLT to either post-OLT day 1 (1.2 ± 37.0 kg vs. 1.2 ± 33.0 kg, p = 0.995) or post OLT day 3 (-0.2 ± 37.0 kg vs. -1.4 ± 35.0 kg, p = 0.70). These data lend support to the notion that the observed differences in renal outcome post-OLT in AKI vs. NO AKI patients cannot be attributed to differences in peri-operative volume status or extent of fluid administration. Moreover, as discussed above, AKI patients on average sustained a weight loss of ~14 kg while awaiting OLT. Indeed, it is noteworthy that SCr decreased despite a large decrease of weight, of which much was likely achieved through fluid removal. Taken together, the observed pre-OLT decrease of SCr among AKI patients would seem to be independent of dilutional factors.

We note the following limitations to our study. First, we were unable to determine the etiology of renal dysfunction in our patients. The distinction between prerenal and renal causes of renal dysfunction pre-OLT may impact on the interpretation and significance of improved renal function as a risk factor for AKI post-OLT.

Third, we used the MDRD formula to determine eGFR. Estimates of renal function in cirrhotic patients that are based on SCr are known to overestimate the true GFR. However, it is important to emphasize that our analysis was limited to the correlation between changes (not absolute levels) of eGFR and the development of AKI following OLT.

Finally, although we included MELD scores in our analysis, our patients came from the pre-MELD era, and risk factors for AKI may differ in the current era, in which patients undergo OLT with more severe renal function. In the last two decades, there have been medical and surgical advances in the management of patients undergoing OLT. These include caval sparing, split liver transplants, diminished usage of veno-veno bypass, shorter anhepatic times, improved anesthetic techniques, and strategies to minimize calcineurin exposure[36-38]. These improvements, while decreasing the risk for AKI during OLT, are counterbalanced by two factors, which simultaneously increase the risk for AKI: transplantation of patients with higher MELD scores, and expansion of the donor pool to include non-heart-beating and "expanded-criteria donors [39-41]." With those caveats in mind, we believe that the impact of pre-operative changes in renal function on the risk for peri-operative OLT AKI is a reflection of the cirrhotic hemodynamic milieu per se, rather than any specific level of hepatic dysfunction, as indicated by the MELD score. In addition, our results may have a more general relevance and extend to surgical procedures other than OLT in patients with end stageliver disease. Analysis of newer databases should help to resolve both of these points.

## Conclusion

In summary, our study is the largest to date on the development of AKI post-OLT using AKIN definitions. Our most important finding is that the absolute level of renal function pre-OLT was not a risk factor for AKI post-OLT. Rather, patients whose renal function declined while awaiting OLT were protected from AKI. This finding may represent true protection, via perhaps ischemic pre-conditioning, or represent a masking of milder forms of AKI, via correction of the cirrhotic milieu and improved renal perfusion. Irrespective of mechanism, the seemingly paradoxical result that declining renal function identifies patients at lesser risk for AKI post-OLT underscores the complex interrelationship between liver and kidney disease. Given the scarcity of organs available for simultaneous liver-kidney transplant, there is a need for additional prospective studies, which might include an analysis of peri-OLT kidney biopsies as part of the study protocol, to dileneate the impact of pre-OLT renal dysfunction following OLT. Our study suggests that not only the etiology but also the course of of pre-OLT renal dysfunction may play a critical role.

## Competing interests

The authors declare that they have no competing interests.

**RESEARCH SUPPORT**: This work was supported by a GRIP (Genzyme Renal Innovations Program) Award from Genzyme, Inc.

**DISCLOSURES**: Jerrold S. Levine, Research funding from Genzyme, Inc.

## Authors' contributions

JII;Principal investigator, participated in design of study, statistical analysis, review, manuscript preparation and data management. Also participated in review of literature, manuscript creation and review. JD; participated data review writing manuscript editing, preparation and statistical review. JSL; participated in study design, Senior investigator statistical analysis, review, editing and preparation of manuscript. All authors read and approved final manuscript and revisions.

## Pre-publication history

The pre-publication history for this paper can be accessed here:

http://www.biomedcentral.com/1471-2369/11/30/prepub

## Supplementary Material

Additional file 1 **Supplemental tables**. Word DOC containing Table S1, S2, S3 and S4 Click here for file
