# Supplementary material for: Risk factors for acute kidney injury following orthotopic liver transplantation: the impact of changes in renal function while patients await transplantation
Source: BMC Nephrol. 2010 Nov 8;11:30. doi: 10.1186/1471-2369-11-30 (PMC2991287; doi:10.1186/1471-2369-11-30)
Supplement: Supplementary file 1 [file 1471-2369-11-30-S1.DOC]

Additional file 1. Table S1. Univariate analysis of measures of renal function in patients with and without AKI (including 11 patients requiring renal replacement therapy on first post-operative day).

Renal functional parameter	NO AKI (n=445)	AKI (n=254)	p value
Iodothalamate clearance (ml/min) (registration)	97 ± 35	104 ± 64	0.29
SCr (mg/dL) (registration)	1.05 ± 0.60	1.06 ± 0.46	0.83
BUN (mg/dL) (registration)	19 ± 18	17 ± 15	0.23
eGFR (ml/min) (registration)	83 ± 31	84 ± 31	0.79
SCr (mg/dL) (pre-operative)	1.19 ± 0.60	1.12 ± 0.53	0.12
BUN (mg/dL) (pre-operative)	19.7 ± 17.0	21.0 ± 16.6	0.38
eGFR (ml/ml) (pre-operative)	76.0 ± 31.0	81.1 ± 33.0	0.036
ΔSCr (mg/dL)	0.13 ± 0.70	0.04 ± 0.390	0.023
ΔBUN (mg/dL)	1.08 ± 23.0	3.0 ± 23.0	0.31
ΔeGFR (ml/min)	-7.4 ± 26.0	–2.7 ± 30.4	0.04
% ΔSCr	19 ± 67	9.3 ± 41	0.014
% ΔBUN	58 ± 170	74 ± 181	0.26
% ΔeGFR	–3.7 ± 45.0	1.8 ± 38.0	0.08
ΔSCr/Δt (mg/dL/day) (mg/dL/day)	0.004 ± 0.034	–0.0005 ± 0.02	0.03
ΔBUN/Δt (mg/dL/day)	0.12 ± 1.30	0.22 ± 1.4	0.138
ΔeGFR/Δt (ml/min/day)	–0.15 ± 1.29	0.01 ± 0.75	0.04

Abbreviations: BUN, blood urea nitrogen; eGFR, estimated glomerular filtration rate; SCr, serum creatinine.

To convert to S.I. units: multiply BUN by 0.357 (mmol/L); multiply SCr by 88.4 (μmol/L).

Additional file 1 Table 1 description: univariate analysis of measurements of renal function in patients with and without AKI. With inclusion of patients requiring dialysis on first post OLT day.

Additional file 1. Table S2. Multivariate logistic regression analysis of risk factors for the development of AKI-1.

Variable	Regression coefficient	SE	p value	OR	95% CI	Coefficient of determination*
Tacrolimus level, day 3	0.232	0.036	0.00001	1.26	1.2-1.3	0.150
Body mass index	0.076	0.025	0.003	1.08	1.03-1.13	0.035
Urine output (cross clamp)	-0.004	0.001	0.002	0.996	0.994-0.99	0.030
Stroke volume (pre-operative)	0.009	0.003	0.007	1.009	1.003-1.016	0.019
Race (non-Caucasian)	0.84	0.293	0.004	2.3	1.3-4.1	0.013
Veno-veno bypass	-0.824	0.29	0.004	0.44	0.25-0.77	0.013
Ascites (pre-operative)	0.66	0.28	0.018	1.94	1.12-3.36	0.013
Serum bicarbonate	-0.077	0.033	0.020	0.92	0.87-0.98	0.011

- The cumulative coefficient of determination for all variables combined is 0.284.

Additional file 1. Table S2. description: multivariate analysis of risk factors for AKI 1

Additional file 1. Table S3. Multivariate logistic regression analysis of risk factors for the development of AKI-2 and AKI-3.

	Regression coefficient	SE	p value	OR	95% CI	Coefficient of determination*
Tacrolimus level, day3	0.298	0.051	0.00001	1.34	1.22-1.49	0.110
SVR (pre-operative)	-0.004	0.001	0.008	0.996	0.993-0.999	0.039
Cyclosporin use	-1.27	0.53	0.017	0.28	0.098-0.797	0.026
% ΔeGFR	0.020	0.008	0.017	1.02	1.004-1.037	0.026
Body mass index	0.14	0.056	0.012	1.15	1.032-1.28	0.023

- The cumulative coefficient of determination for all variables combined is 0.224.

Additional file 1. Table S3. description: Multivariate analysis of risk factors for the development of AKI-2 and AKI-3

Additional file1. Table S4. Univariate analysis of measures of renal function in patients with and without AKI, stratified according to class of AKI.

	No AKI (n=445)	AKI-1 (n=178)	AKI-2 (n=45)	AKI-3 (n=20)	p value
Iodothalamate clearance (ml/min) (registration)	97 ± 35	95 ± 31	102 ± 33	164 ± 155	0.09
SCr (mg/dL) (registration)	1.05 ± 0.60	1.07 ± 0.41	1.00 ± 0.45	1.02 ± 0.50	0.40
BUN (mg/dL) (registration)	19.7 ± 17.0	20.0 ± 14.0	14.6 ± 11.2	15.7 ± 8.1	0.26
eGFR (ml/min) (registration)	83.0 ± 31.0	82.1 ± 33.2	87.5 ±24.7	93.3 ± 32.0	0.09
SCr (mg/dL) (pre-operative)	1.19 ± 0.60	1.13 ± 0.43	1.00 ± 0.37	0.98 ± 0.48	0.03
BUN (mg/dL) (pre-operative)	19.7 ± 17.0	18.0 ± 15.1	21.4 ± 20.1	18.5 ± 16.5	0.24
eGFR (ml/min) (pre-operative)	76.0 ± 31.0	77.3 ± 29.4	88.0 ± 29.9	103.6 ± 52.0	0.01
ΔSCr (mg/dL)	0.13 ± 0.70	0.05 ± 0.41	0.00 ± 0.37	-0.04 ± 0.22	0.06
ΔBUN (mg/dL)	1.1 ± 23.0	2.1 ± 21.0	6.8 ± 23.2	2.8 ± 20.3	0.14
ΔeGFR (ml/min)	-7.4 ± 26	-4.8 ± 29	0.5 ± 30.6	10.3 ± 42.1	0.04
% ΔSCr	19 ± 67	11 ± 43	5.4 ± 34.4	–2.6 ± 23	0.04
% ΔBUN	58 ± 170	69 ±1 81	90 ± 179	62 ± 191	0.09
% ΔeGFR	-3.7 ± 45	0.3 ± 37	6.1 ± 41	11.5 ± 39	0.04
$\Delta SCr/\Delta t_{wait}$ (mg/dL/day)	0.004 ± 0.034	–0.0013 ± 0.017	–0.0015 ± 0.008	–0.0018 ± 0.006	0.02
$\Delta BUN/\Delta t_{wait}$ (mg/dL/day)	0.12 ± 1.30	0.17 ± 0.95	0.18 ± 0.86	0.13 ± 0.55	0.17
$\Delta eGFR/\Delta t_{wait}$ (ml/min/day)	–0.15 ± 1.29	–0.0003 ± 0.68	0.09 ± 0.43	0.28 ± 0.84	0.01

Abbreviations: BUN, blood urea nitrogen; eGFR, estimated glomerular filtration rate; SCr, serum creatinine.

To convert to S.I. units: multiply BUN by 0.357 (mmol/L); multiply SCr by 88.4 (μmol/L).

Additional file 1. Table S4. Univariate analysis of measures of renal function in patients with and without AKI, stratified according to class of AKI.
